# Impact of the *ActTeens Program* on physical activity and fitness in adolescents: a cluster randomized controlled trial

**DOI:** 10.1186/s12887-024-04922-9

**Published:** 2024-07-11

**Authors:** Géssika Castilho dos Santos, Thais Maria de Souza Silva, Jadson Marcio da Silva, Rodrigo de Oliveira Barbosa, Sarah G. Kennedy, David R. Lubans, Antonio Stabelini Neto

**Affiliations:** 1 Health Science Center, Universidade Estadual do Norte do Paraná, Jacarezinho, PR Brazil; 2 Post-Graduate Program in Physical Education Associate UEM/UEL, Universidade Estadual de Londrina, Londrina, PR Brazil; 3 Health and Physical Education, School of Health Sciences, Western Sydney University, Kingswood, NSW Australia; 4 Centre for Active Living and Learning, College of Human and Social Futures, University of Newcastle, Callaghan, NSW Australia; 5 Hunter Medical Research Institute, New Lambton Heights, NSW Australia; 6 Faculty of Sport and Health Sciences, University of Jyväskylä, Jyväskylä, Finland

**Keywords:** Exercise, Physical Education and Training, Youth

## Abstract

**Background:**

The aim of our study was to evaluate the impact of the ActTeens Program on physical activity and health-related physical fitness among adolescents in Brazil.

**Methods:**

The “ActTeens Program” was conducted using a cluster-randomized controlled trial during 24-week school term. The sample consisted of 317 adolescents (52.7% girls; 13.61 ± 0.70 years) from four secondary schools that were randomly assigned to intervention group (*N* = 169) or control group (*N* = 148). This school-based physical activity (PA) intervention involved two components: (i) structured physical activity sessions delivered within physical education (PE) and (ii) healthy lifestyle guidance (mHealth). The primary outcome was PA assessed using Physical Activity Questionnaire for Adolescents (PAQ-A); secondary outcomes included muscular (MF) and cardiorespiratory fitness (CRF) assessed using 90-push-up, handgrip dynamometer, standing long jump, and 20 m PACER shuttle run test. Assessments were conducted at baseline, 12- and 24-week. Intervention effects were assessed using linear mixed models (LMM).

**Results:**

For the primary outcome (PA), no significant group-by-time effects were observed for physical education based-PA (0.3 score; 95%CI: -0.1; 0.6; and − 0.01 score; 95%CI: -0.03; 0.03, at 12-wk and 24-wk respectively) and total PA (-0.02 score; 95%CI: -0.2; 0.2; and − 0.01score; 95%CI: -0.2; 0.2, at 12 and 24 weeks respectively). After 24 weeks, we observed a significant group by time effects for lower body muscular fitness (12.9 cm; 95%CI, 3.2 to 22.2).

**Conclusion:**

The implementation of aerobic and muscle-strengthening exercises used in the ActTeens intervention did not lead to improvements in physical activity. The intervention resulted in improved lower body muscular fitness, however, we found no significant differences for upper body muscular and cardiorespiratory fitness.

**Supplementary Information:**

The online version contains supplementary material available at 10.1186/s12887-024-04922-9.

## Background

A physically active lifestyle is associated with numerous physical, psychological, and cognitive health benefits for adolescents, such as improved cardiorespiratory and muscular fitness, reduced symptoms of depression, and enhanced cognitive function [1–3]. In addition, physical activity (PA) is a protective factor against the occurrence of various non-communicable diseases (e.g. hypertension, diabetes mellitus, cardiovascular disease, cancer) throughout life [1, 2]. In order to achieve these health benefits, it is recommended that children and adolescents participate in 60 min/day of moderate-to-vigorous physical activity (MVPA), as well as muscle and bone strengthening activities [e.g., resistance training (RT)] three days a week [1, 4].

Although there is sufficient evidence of the effects of PA on health, data suggests that less than 20% of school-age adolescents across the globe are physically active [5]. According to a recent survey by the Report Card Brazil project, only 29.9% Brazilian adolescents engage in adequate MVPA, with lower levels among females compared with males (29.5% vs. 44.6%, respectively) [6]. Of additional concern, systematic reviews have reported a temporal decline in PA [7] and health-related physical fitness (PF) levels (cardiorespiratory and muscular) [8, 9], which indicates that today’s children and adolescents have lower levels of PA, CRF and MF than their peers from previous generations [7–9].

Importantly, PF in childhood and adolescence has been considered a powerful predictor of health later in life, as behaviours developed in childhood are often carried on into adulthood [10] and adequate levels of PA are essential for the development and maintenance of health-related PF [11]. This is apparent as consistent findings have shown that a high level of MVPA was associated with better aerobic and muscular fitness in adolescents [12, 13], since PA and PF positively influencing each other [14]. For this reason, creating opportunities for adolescents to participate in PA that improve their health is of considerable importance and should be one of the public health priorities.

Schools are an ideal setting to promote and offer opportunities for PA among adolescents, as they spend a large proportion of their time at school [15]. Physical Education (PE) classes can help adolescents to consolidate active lifestyle habits that will last a lifetime [15, 16], through games, sports and non-traditional PA (e.g. resistance training, yoga, etc.). The school and particularly PE classes, can provide students with the knowledge, skills, and attitudes to adopt and maintain physically active lifestyles [17].

However, findings from school-based PA interventions have been inconsistent, with recent review reporting non-significant changes in objectively measured PA across the school day [18]. Multicomponent interventions (i.e., comprehensive school-based PA programs) appear to be more successful than single-component interventions [18]. This multicomponent approach includes offering PA opportunities before, during, and after school during PE, recess breaks, classroom-based activities, active transportation to/from school, and sports participation [19]. Moreover, social support provided by family, which is a critical factor that influences children and adolescents attitude towards PA should be considered in adolescent intervention efforts [20].

Digital programs (eHealth and mHealth) have emerged as a potentially efficacious strategy for promoting PA beyond the school setting (e.g., home, park, leisure time, and transportation) [21]. It is important to emphasize that during COVID-19 pandemic, several mHealth-based interventions were implemented as they offered a potential solution to social distancing and lockdown (home confinement) [22]. Prior research [18] has been shown the potential of eHealth and mHealth interventions for changing adolescents’ activity behaviours in the short -term, particularly when integrated with other intervention components (e.g., school-based environmental changes).

Multi-component PA interventions can improve PA levels [23, 24], cardiorespiratory [24, 25] and muscular fitness [26, 27], skill competency [26, 27] and reduction in screen time [27, 28] in adolescents. In Brazil, previous school-based programs [23, 29, 30] have been developed with a focus on promoting active behaviour in teens. However, these programs have only included school-based strategies (i.e., no external support for families) and have predominantly focused on the aerobic component of youth PA guidelines. Thus, the aim of the current study was to evaluate the impact of ActTeens Program on physical activity and fitness among adolescents in Brazil. We hypothesized that the ActTeens Program would increase physical activity and improve health-related fitness outcomes in adolescents.

## Methods

### Study design and participants

We evaluated the ActTeens Program using a 24-week cluster randomized controlled trial (RCT) that adhered to CONSORT recommendations. ActTeens included multiple components to promote PA in adolescents. The trial was approved by the human research ethics committee of the States University of Northern of Parana, Brazil (nº 4.452.513) and registered in the protocol of Clinical Trials (NCT05070377, 7/10/2021).

The intervention was conducted in a school environment during one school year (February to December). Data were collected at baseline [March 2022], post-12 weeks [July 2022] and post- 24 weeks [November 2022]. PA measures were collected at all three-time points and PF collected at (baseline and 24-weeks). Secondary public schools in Jacarezinho City, including students aged 12–15 years (i.e., grades 8 and 9) were eligible to participate. The study protocol that described the study rationale, design, and measurement has been published previously [31]. The sample size calculation was based on detecting changes in the primary outcome (PA). The calculation assumed an effect size of 0.10, power of 95%, a 5% level of significance and correlation was assumed as 0.03. Considering an assumed attrition of 20%, a minimum of 280 students was required. Considering that each school class has approximately 25 students, 6 classes were randomized from each group, totalling 140 students in each condition (intervention and control).

**Fig. 1:**
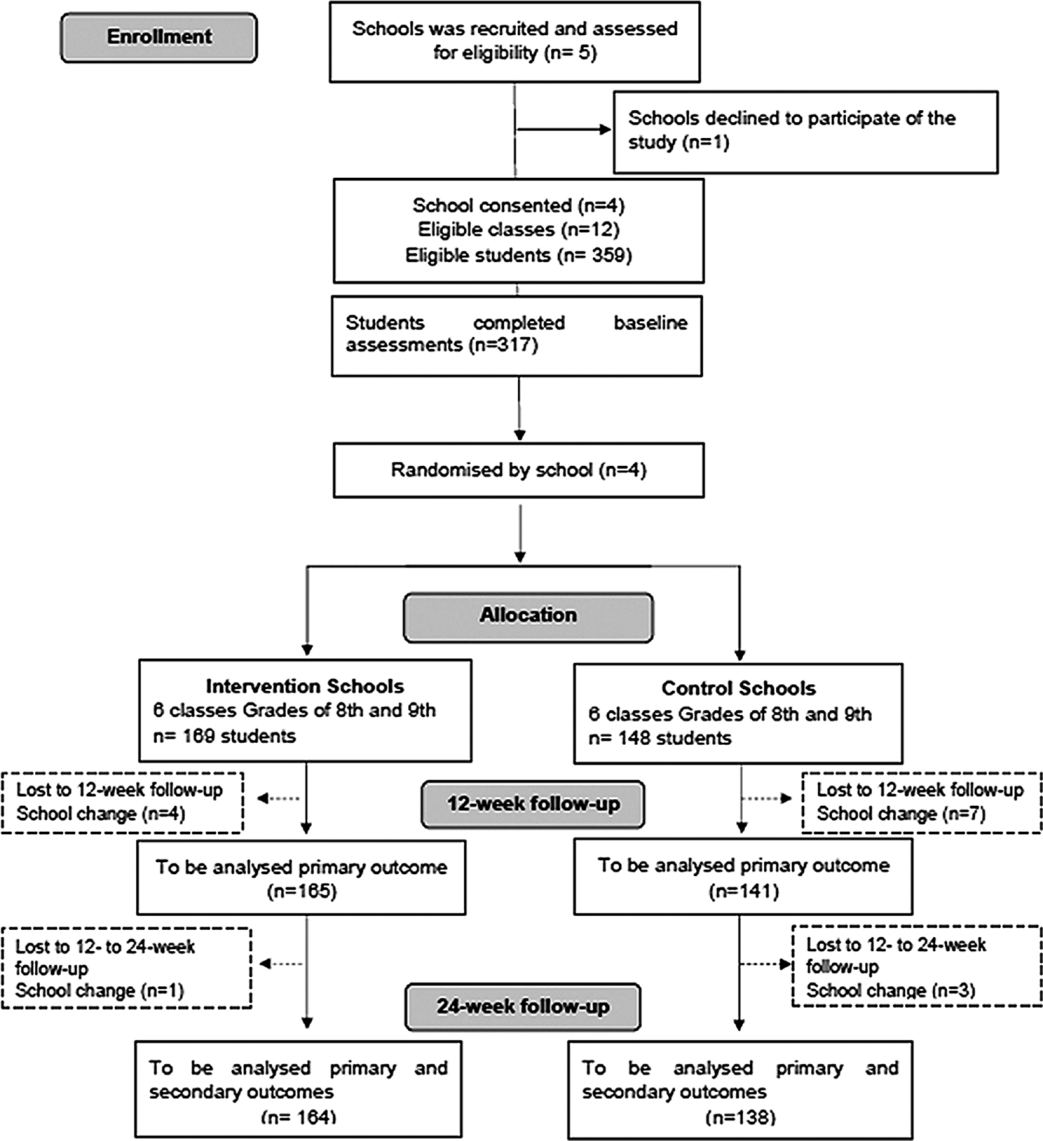
Flow diagram throughout the course of the study

Four schools were randomized to either a control (CG) or an intervention (IG) group by an independent researcher using a computer-based random number generator. A total of 317 students from secondary public schools were assessed for eligibility and agreed to participate in the study. Both parents and adolescents provided written informed consent to participate. During the intervention period, fifteen participants withdrew from the study for personal reasons related to switching schools (see Fig. 1; CONSORT flow diagram).

### ActTeens Program

The PA program was implemented over 24-weeks (i.e., term 1 and 2/ 12 weeks of intervention; holiday of 2 weeks at the end of Semester 1; and term 3 and 4/ 12 weeks of intervention) and included two components: (1) structured PA sessions delivered within PE classes; and (2) healthy lifestyle guidance via mHealth. The strategies were guided by self-determination [32] and social cognitive theories [33].

The structured PA sessions were implemented over 24 weeks and delivered within PE lessons, twice a week, 20 min per lesson. These sessions were adapted from the Resistance Training for Teens (RT4T) program [26]. RT4T is an Australian program designed to improve muscular fitness and provide adolescents with the knowledge, motivation, skills, and confidence to engage in resistance training. The PA sessions were designed to satisfy participants’ basic psychological needs for autonomy, competence, and relatedness, and to promote autonomous motivation and self-efficacy for PA. The structured PA sessions consisted of a combination of muscle-strengthening and aerobic exercise [26]. The session followed a specific format including i) movement-based games and dynamic stretching warm-up (3 min); structured PA (15 min); and cool-down including static stretching (2 min). In each structured PA session, participants were able to choose their own groups (small groups of 4–5 people) and were given autonomy to choose the sequence of exercises they would like to perform (4–5 exercises), within a variety of cards, incorporating aerobic and resistance exercises. At the end of the structured PA session, the adolescents were asked to report the intensity immediately after the last exercise of the session using Borg’s rating of perceived exertion scale.

Weekly messages (video or infographic) were sent via the WhatsApp^®^ app (mHeath) to promote healthy behavior, to both adolescents (twice a week) and their parents (twice a month) (i.e. social support). The messages were specific to promote and encourage active behavior and provide guidance on healthy eating habits. Twenty-four messages were sent. The content of the messages was focused on educational/counselling and practice tips about PA, goal setting and challenges, as well about a healthy lifestyle (PA associated with healthy eating and sedentary behavior) (see supplementary Figs. 1, 2 and 3).

Adolescents and their parents in the control group received the same messages about healthy behavior via the WhatsApp^®^ app as the intervention group. However, the adolescents in the control condition continued with their PE classes as usual.

### Measures and data collection

All assessments were conducted in the study schools by trained research assistants, who were blinded to group allocation at all-time points (baseline, mid and post-intervention). The research assistants provided a brief verbal description and demonstration of each fitness test before the start. Self-report information was assessed using specific questionnaires. Anthropometric assessments were conducted by two researchers of both sexes.

### Primary outcome: physical activity

PA was assessed using the ‘Physical Activity Questionnaire for Adolescents’ (PAQ-A), that has been translated and validated for Brazilian adolescents [34]. The PAQ-A is a self-administered, 7-day recall questionnaire, that assesses participation in different PA (during PE classes, lunch break, after school, in the evenings and at weekends). In this study, we used the question “In the last 7 days, during your PE classes, how often were you very active (playing hard, running, jumping, throwing)?” to assess PA during PE classes and the total questionnaire score to assess total PA.

### Secondary outcomes

Muscular fitness. Upper body muscular strength was assessed using 90- push-up test [35] and handgrip dynamometer (Jamar Technologies) [36]. In the 90- push-up test, the participant should lower their body until a 90-degree angle is formed at the elbow before pushing back up, using a cadence of 40 beats per minute. The test was concluded when the participant either fails to do a push-up in the angle required on two non-consecutive repetitions (warning verbalized by an assessor, repetitions counted), fails to maintain movement in time with the metronome, fails to maintain appropriate technique (back straight) or on the volitional failure of the test [35]. In the handgrip test, the participant held the dynamometer with the hand and positioned the arm at right angles and the elbow on the side of the body. When ready the subject squeezed the dynamometer with maximum isometric effort, which was maintained for about 5 s [36]. The average of the best scores of each hand was adopted as the muscular fitness indicator. The lower body muscular strength was measured using the standing long jump test [37], which has excellent validity and reliability [38, 39] in adolescent population. From a standing position behind a line marked at zero centimeters, participants perform a maximal long jump taking off and landing with two feet, simultaneously. The test was performed twice, with adequate rest between attempts, with the maximal distance jumped recorded as the participant’s final score.

Cardiorespiratory fitness was assessed using the 20-m PACER shuttle run test and administered following standardized procedures [40]. A 20-m course was set up indoors on a hard surface with students instructed to run back and forth between two lines following an accompanying audio file. Test administrators provided verbal encouragement to participants to maximize motivation. The test was ended when the participant fails to complete two consecutive laps in the allotted time or voluntarily dropped out due to fatigue. The total number of laps was registered and used as cardiorespiratory fitness outcome.

### Demographic variables

Adolescents were asked about their age, gender and parent education level. Information on sports practice among adolescents was obtained by one question: “Do you practice any sports?” in a dichotomous manner (yes or no). Maturational status was estimated through the evaluation of somatic maturation by determining the distance in years of the individual from the baseline peak height velocity (PHV) using sex-specific mathematical models based on measures of height, age, and sex [41].

Body mass was measured to the nearest 0.1 kg in light clothing without shoes using a portable digital scale (Welmy^®^, Santa Bárbara do Oeste, São Paulo, Brazil) and height was measured using a portable stadiometer (Welmy^®^, Santa Bárbara do Oeste, São Paulo, Brazil). Body mass index (BMI) was calculated according to ACSM [42]. Waist circumference was measured twice at the midpoint between the last rib and the iliac crest using steel tape [42].

### Process evaluation

A detailed process evaluation was conducted and included the following: (1) intervention implementation (e.g., number of structured PA sessions that were delivered); (2) reach (number of students who agreed to participate in the program); (3) retention rate (referring to the 24 weeks follow-up); (4) frequency (student participation in structured PA sessions).

### Statistical analysis

Descriptive statistics included frequency and percentages for categorical variables, and mean with standard deviation (SD) or 95% confidence intervals (95% CI) for continuous variables. Chi-square and t-tests were used to identify the possible differences between participants within the intervention and control groups at baseline. Analyses of the primary and secondary outcomes were conducted using linear mixed models in IBM SPSS Statistics for Windows (version 25.0; 2010 SPSS Inc, IBM Company, Armonk, NY), with alpha levels set at *P* < .05. Models assessed the effect of treatment (intervention or control), time (baseline, 12 weeks [only PA outcome], and 24 weeks), and the group–time interaction. Time and treatment were included as fixed factors, with school class included as a random effect [43].

The models were adjusted for the following variables: sex, PHV, sports practice and BMI. A sensitivity analyses was conducted in the last observation carried forward analysis (see supplementary Tables 1 and 2). Differences at baseline between completers and those who dropped out of the study were examined using independent-samples t-tests (see supplementary Table 3).

## Results

A total of 317 adolescents (52.7% females; age 13.61 ± 0.7 years) completed the baseline assessments. The baseline characteristics of the participants are presented in Table 1. Post-12-week and post 24-week assessments were completed by 96.8% and 95.2% students, respectively.

**Table 1:** Baseline characteristics of the study sample

Variables	Intervention Group (*n* = 169)	Control Group (*n* = 148)	Total (*n* = 317)
Age (years), mean(SD)	13.5 (0.7)	13.6 (0.6)^*^	13.6 (0.7)
Female n, (%)	86 (50.9)	81 (54.)	167 (52.7)
BMI (Kg/m^2^), mean(SD)	20.9 (4.9)	20.8 (4.5)	20.7 (4.8)
WC (cm), mean(SD)	71.1 (11.9)	68.5 (9.8)	67.9 (9.8)
PHV (years), mean(SD)	0.6 (0.8)	0.6 (0.8)	0.6 (0.8)
Habitual PA practice, score	2.2 (0.6)	2.1 (0.6)	2.2 (0.6)
Sports practice, n (%)			
Yes	21 (13.4)	33 (22.9)^*^	54(17.9)
No	136 (86.6)	111(77.1)	247 (82.1)
Mother’s educational level, n (%)			
Secondary education incomplete	8 (10.6)	9 (17.7)	17 (13.5)
Secondary education complete	10 (12.7)	3 (5.9)	13 (10.3)
High school	36 (53.4)	25 (49)	61 (48.4)
Graduated	17 (22.7)	7 (13.7)	24 (19.0)
Not informated	4 (5.3)	7 (13.7)	11 (8.7)
Father’s educational level, n (%)			
Secondary education incomplete	12 (17.6)	12 (24)	24 (20.3)
Secondary education complete	11(16.2)	9 (18)	20 (17)
High school	29 (42.7)	13 26)	42 (35.6)
Graduated	9 (13.2)	4 (8)	13 (11)
Not Informated	7 (10.3)	11 (11)	18 (15.3)

SD, standard deviation; n, sample; BMI: Body mass index; WC: waist circumference; PHV: Peak height velocity, PA: Physical activity. * *P* < .05 significant difference between groups

Changes in adolescents’ physical education-based PA and total PA across different time points (baseline, 12 and 24 weeks) by groups are presented in Table 2. We observed a significant increase in PE-based PA from baseline to 12- and 24-weeks in both groups. However, there were no group-by-time effects for PA.

For muscular fitness, there was a significant group by time effect for lower body muscular fitness (mean difference: 12.9 centimeters, 95% CI, 3.2 to 22.2). However, no group-by-time effect effects were found for hand grip strength, push-ups, and cardiorespiratory fitness.

**Table 2:** ActTeens Program effects on physical activity outcomes between the groups with intention-to-treat analysis

Measure	Group	Baseline^a^	*n*	12 weeks^a^	*n*	Time, *P*^b^	12 weeks Adj. Diff. in Change^c^	Group-time, *P*^d^	24 weeks^a^	*n*	Time, *P*^b^	24 weeks Adj. Diff. in Change^c^	Group-time, *P*^d^
Score PA, PE	INT	2.5(2.3; 2.6)	157	3.4(3.2; 3.6)	142	< 0.001	0.3 (-0.1; 0.6)	0.12	3.3(3.1 3.6)	137	< 0.001	− 0.01(-0.3; 0.3)	0.94
	CON	2.3(2.1; 2.4)	143	3.0(2.7; 3.2)	113	< 0.001			3.2(2.9; 3.4)	115	< 0.001		
Score PA, total	INT	2.2(2.1; 2.3)	158	2.4(2.3; 2.5)	145	0.08	-0.02 (-0.2;0.2)	0.81	2.3(2.1; 2.4)	142	1.0	− 0.01(-0.2; 0.2)	0.91
	CON	2.1(2.0; 2.2)	144	2.3(2.2; 2.4)	113	0.06			2.2(2.0; 2.3)	117	1.0		

^a^Mean (95% CI). ^b^Within-group change over time (baseline). ^c^Adjusted mean difference (95% CI) between the intervention and the control group at the specified time point. ^c^Group-by-time interaction from linear mixed model that included baseline and the specified time point

CON, control; INT, intervention; PA, physical activity; PE, physical education

**Table 3:** ActTeens Program effects on physical fitness outcomes between the groups with intention-to-treat analysis

Measure	Group	Baseline^a^	*n*	24 weeks^a^	*n*	Time, *P*^b^	24 weeks Adj. Diff. in Change^c^	Group-by-time, *P*^d^
Push-ups, reps	INT	4.7 (3.6; 5.8)	145	6.2(4.8; 7.6)	122	0.06	0.7 (-1.4; 3.0)	0.49
	CON	3.7 (2.7; 4.7)	128	4.4 (3.2; 5.7)	111	0.37		
Handgrip strength, kg	INT	25.1 (24.1; 26.2)	153	26.6 (25.2; 28.0)	132	0.05	0.8 (− 1.2; 2.9)	0.41
	CON	25.9 (24.9; 26.9)	137	26.5 (25.2; 27.7)	117	0.45		
Standing long jump, cm	INT	127.3 (122.6;132.0)	152	135.9 (130.4;141.4)	125	0.01	12.9 (3.2;22.2)	0.007
	CON	135.9 (131.3;140.2)	136	131.6 (126.3; 136.8)	116	0.19		
20 m shuttle run test, laps	INT	25.6 (23.0; 28.2)	142	29.7 (26.7; 32.7)	105	0.01	-1.2 (-6.0; 3.4)	0.595
	CON	21.3 (19.0; 23.6)	124	26.7 (23.7; 29.6)	107	0.002		

^a^Mean (95% CI). ^b^Within-group change over time (baseline). ^c^Adjusted mean difference (95% CI) between the intervention and the control group at the specified time point. ^c^Group-by-time interaction from linear mixed model that included baseline and the specified time point

CON, control; INT, intervention; PA, physical activity; PE, physical education

In addition, similar findings were observed in last observation carried forward (see supplementary Tables 1 and 2, last observation carried forward analysis of primary and secondary outcomes) sensitivity analyses when compared with intention-to-treat. A difference of note, however, is that the last observation carried forward analysis indicated a significant group–time effect for PE based-PA score at 12 weeks (mean difference 0.34 score; 95% CI, 0.0 to 0.6).

Overall, the structured sessions were delivered in 83.3% of PE lessons and the reach of the ActTeens intervention was 88.3% (317/359 eligible adolescents) with a retention rate of 95.3% (302/317). Furthermore, in the IG, 79 and 57 adolescents had an attendance above of 75% at 12- and 24-week, respectively.

## Discussion

The current study aimed to evaluate the effects of the ActTeens Program on PA and PF in adolescents. The findings indicated that the current school-based intervention did not increase PA. However, we observed a significant group by time effect for muscular fitness at 24-weeks.

ActTeens’ intervention was not successful in increasing PA outcomes. Promoting active behavior is a complex path, Beets and collaborators [44] developed the theory of expanded, extended, and enhanced opportunities (TEO), that suggests that to increase adolescent’s PA three mechanisms are needed: expansion (i.e., adding new opportunities), extension (i.e., lengthening time currently allocated for PA opportunities), and the enhancement (i.e., modifying an existing PA opportunity). According to these authors, the TEO takes a more pragmatic approach that gives increased emphasis to PA opportunities and considers how these opportunities can be modified to increase PA engagement.

The findings of the current study in relation to PA are contrary to results from previous school-based multi-component interventions [23, 45] conducted during school time with adolescents, where the strategies were applied in PE classes, daily recess and extracurricular activity. The different results may be partially explained by the fact that the ActTeens intervention offered structured PA opportunities only within PE classes. In accordance with our justification, Harris and Cale [17] showed that modifying the PE curriculum alone does not always lead to improvements in PA. Another reason for our results may be due by the lack of strategies for promoting PA practice outside the school context, which can explain the null effects for PA in our intervention. For example, CHAMPS program [45] strategies were included in PE classes (three times a week for 45 min each), daily recess (5 d/week for 15 min), text messages on healthy tips (bi-weekly) and after-school (45 min of MVPA 2 d/week), which increased in the time spent in MPA, VPA, and MVPA. This information suggested that it is necessary to implement opportunities to PA practice in other contexts rather than PE.

We included an mHealth component in the ActTeens intervention to stimulate PA among adolescents beyond the school setting [31]. This included sending messages about the importance and benefits of adopting a healthy lifestyle for adolescents. Messages were also delivered to parents via WhatsApp^®^ to inform them about the importance of family support in the behavior change of adolescents. However, the educational/counselling actions did not promote an increase in PA scores. A possible explanation is that not all individuals pay attention to the messages, since that, in the current study, around 57.9% (data not shown) of adolescents answered that they rarely/never read the messages received. Furthermore, it is worth highlighting that adolescents in both groups received the mHealth intervention and because of this we were unable to analyze the effectiveness of this component.

Our null findings may also be attributable to the measurement of PA using a questionnaire. Although it has been previously validated and reliable, it provides a subjective measurement of PA [34]. In addition, it reports general PA but does not differentiate the intensities of the PA. Therefore, adolescents may be more active/participative, but they are not able to differentiate how much more through the PAQ-A questionnaire. Consequently, future research needs to analyze differences in the level of PA practiced using a device-based measure such as accelerometry.

Regarding PF outcomes, the results showed that the intervention led to a significant group-time effect for standing long jump (12.9 centimeters, 95% CI, 3.2 to 22.2) (Table 3). Yet, no effect was found for handgrip and push-ups. These findings for lower body muscular fitness may be due to the inclusion of exercises that required lower body power (e.g., jumps squats, jumping lunges, jump role), which also may have improved fitness. While for upper body muscular fitness are likely due to the specificity of the exercise training chosen by adolescents during the intervention, where they generally excluded exercises such as push-up and burpee. In addition, no specific exercises were included to improve upper member isometric strength (i.e. handgrip).

Both groups increased their CRF, and the group–time effect was not significant. Our findings are similar to results from a cluster randomized controlled study that examined the effect of a school-based intervention on CRF among Brazilian students [46]. A possible reason for the increase of laps in CG adolescents may be explained by out-of-school sports participation, which was not asked post-intervention.

These findings are promising because health-related PF have been considered a protective predictor against the development of cardiometabolic diseases [47]. Like this, the ActTeens intervention highlights the potential for 20 min exercises within PE classes to improve lower body muscular fitness; it is important to highlight that most PA programs aiming to improve MF (e.g. resistance training) involved substantial space and equipment requirements, and are conducted outside the school environment. Considering points above, the ActTeens Program is able help to overcome these barriers because ActTeens can be implemented within PE classes using an inexpensive minimal equipment (e.g. sets of circuit cards, fitness equipment packs and body weight exercises only), suggesting that the intervention has the potential to be scaled-up and disseminated from different schools including areas of socioeconomic disadvantage, particularly in low-middle income countries.

The strengths of this study include the cluster RCT design; high retention rate; the sample size with sufficient statistical power; and the use of robust analysis. In addition, the strategies proposed in the ActTeens program are scalable, since the implementation of the structured PA sessions did not require total modification in the curriculum content of PE classes. However, there are some limitations that should be mentioned. These include the lack of accurate control and monitoring (e.g. heart rate) over the intensity of structured activities delivered within PE lessons. The subjective measure of PA also represents a limitation, which have well-known disadvantages such as recall bias. The messages about healthy behavior were read by less than half (42.1%) of the adolescents, in addition, the effectiveness of this intervention component cannot be verified since both groups received the same mHealth intervention.

## Conclusion

The ActTeens program, which included structured PA session into PE had a positive effect on adolescents’ lower body MF after intervention. These findings suggest that the inclusion of combined activities (aerobic and muscle-strengthening) in PE lessons can be done in Brazilian public schools without modifying the PE classes’ curriculum. The proposed strategies can be used in further interventions and public policies focused on promoting MF among adolescents. Nevertheless, further investigation underscores the need for a scale-up evaluation of ActTeens Program, with future directions for this research including a large-scale dissemination.

### Practical implications

- The structured PA session implemented by “ActTeens Program” is an effective strategy for promoting lower body muscular fitness, and applicable as practical activities last 20 min without radically modify the curriculum content of PE classes.
- Schools play an important role in adolescents’ health. So, these tips about ActTeens structured session should support teachers and school managers on how to create opportunity for students to improve health-related physical fitness.

### Electronic supplementary material

Below is the link to the electronic supplementary material.

Supplementary Material 1

Supplementary Material 2

Supplementary Material 3

Supplementary Material 4

## Data Availability

The dataset of the present study is available upon request from the corresponding author.
