# Supplementary material for: Impact of the ActTeens Program on physical activity and fitness in adolescents: a cluster randomized controlled trial
Source: BMC Pediatr. 2024 Jul 11;24:447. doi: 10.1186/s12887-024-04922-9 (PMC11238359; doi:10.1186/s12887-024-04922-9)
Supplement: Supplementary file 1 — Supplementary Material 1 [file 12887_2024_4922_MOESM1_ESM.docx]

| **Measure** | **Group** | **Baseline^a^** | **n** | **12 weeks^a^** | **n** | **Time, P^b^** | **12 weeks**  **Adj. Diff.**  **in Change^c^** | **Group-time, P^d^** | **24 weeks^a^** | **n** | **Time, P^b^** | **24 weeks**  **Adj. Diff.**  **in Change^c^** | **Group-time, P^d^** |
| --- | --- | --- | --- | --- | --- | --- | --- | --- | --- | --- | --- | --- | --- |
| Score PA, PE | INT | 2.5(2.3; 2.6) | 157 | 3.3(3.1; 3.5) | 157 | <0.001 | 0.34 (0.0; 0.6) | 0.04 | 3.3(3.1; 3.5) | 157 | <0.001 | 0.05(-0.2; 0.3) | 0.76 |
|  | CON | 2.(2.1; 2.4) | 143 | 2.7(2.5; 2.9) | 143 | 0.01 |  |  | 3.0(2.8; 3.2) | 143 | <0.001 |  |  |
| Score PA, total | INT | 2.2(2.1; 2.3) | 158 | 2.4(2.2; 2.5) | 158 | 0.14 | -0.00 (-0.2;0.2) | 0.99 | 2.2(2.1; 2.3) | 158 | 1.0 | -0.03(-0.2; 0.1) | 0.78 |
|  | CON | 2.1(2.0; 2.2) | 144 | 2.3(2.1; 2.4) | 144 | 0.16 |  |  | 2.1(2.0; 2.3) | 144 | 1.0 |  |  |

Supplemental Table I- Last observation carried forward analysis of primary outcomes.

^a^Mean (95% CI).

^b^Within-group change over time (baseline)

cAdjusted mean difference (95% CI) between the intervention and the control group at the specified time point.

^c^Group–time interaction from linear mixed model that included baseline and the specified time point.

CON, control; INT, intervention; PA, physical activity; PE, physical education.
