# Supplementary material for: Impact of the ActTeens Program on physical activity and fitness in adolescents: a cluster randomized controlled trial
Source: BMC Pediatr. 2024 Jul 11;24:447. doi: 10.1186/s12887-024-04922-9 (PMC11238359; doi:10.1186/s12887-024-04922-9)
Supplement: Supplementary file 2 — Supplementary Material 2 [file 12887_2024_4922_MOESM2_ESM.docx]

Supplemental Table II - Last observation carried forward analysis of secondary outcomes.

| **Measure** | **Group** | **Baseline^a^** | **n** | **24 weeks^a^** | **n** | **Time, P^b^** | **24 weeks**  **Adj. Diff.**  **in Change^c^** | **Group-time, P^d^** |
| --- | --- | --- | --- | --- | --- | --- | --- | --- |
| Push-ups, rep | INT | 4.62 (3.52; 5.73) | 145 | 5.65 (4.41; 6.90) | 145 | 0.17 | 0.3 (-1.7; 2.4) | 0.74 |
|  | CON | 3.94 (2.92; 4.96) | 128 | 4.62 (3.52; 5.73) | 128 | 0.35 |  |  |
| Handgrip strength, kg | INT | 25.63 (24.61; 26.66) | 153 | 26.28 (25.19; 27.36) | 153 | 0.32 | -0.04 (- 1.8; 1.8) | 0.96 |
|  | CON | 26.11 (25.08; 27.14) | 137 | 26.71 (25.61; 27.81) | 137 | 0.37 |  |  |
| Standing Long Jump, cm | INT | 128.78 (124.34;133.21) | 152 | 135.18 (130.58;139.78) | 152 | 0.03 | 9.33 (0.9;17.6) | 0.03 |
|  | CON | 136.80 (131.94;141.66) | 136 | 133.88 (128.84; 138.93) | 136 | 0.34 |  |  |
| Laps | INT | 25.40 (22.87; 27.83) | 142 | 28.99 (26.08; 31.90) | 142 | 0.03 | -1.06 (-5.5; 3.4) | 0.64 |
|  | CON | 21.22 (18.93; 23.52) | 124 | 25.87 (23.30; 28.45) | 124 | 0.004 |  |  |

^a^Mean (95% CI).

^b^Within-group change over time (baseline)

cAdjusted mean difference (95% CI) between the intervention and the control group at the specified time point.

^c^Group–time interaction from linear mixed model that included baseline and the specified time point.

CON, control; INT, intervention; REP, repetition ; KG, kilogram; cm, centimeter.
