# Supplementary material for: Impact of the ActTeens Program on physical activity and fitness in adolescents: a cluster randomized controlled trial
Source: BMC Pediatr. 2024 Jul 11;24:447. doi: 10.1186/s12887-024-04922-9 (PMC11238359; doi:10.1186/s12887-024-04922-9)
Supplement: Supplementary file 3 — Supplementary Material 3 [file 12887_2024_4922_MOESM3_ESM.docx]

Supplemental Table III - Baseline characteristics of the study completers’ and dropout.

| **Variables** | **All Participants**  (n=302) | **Dropout**  (n=15) | **p** |
| --- | --- | --- | --- |
| Age (years), mean(SD) | 13.55 (0.70) | 13.71 (0.97) | 0.60 |
| Female n, (%) | 159 (52.6) | 8 (53.3) |  |
| BMI (Kg/m^2^), mean(SD) | 20.80 (4.71) | 21.21 (4.21) | 0.75 |
| WC (cm), mean(SD) | 69.27 (10.54) | 70.53 (8.31) | 0.62 |
| PHV (years), mean(SD) | 0.63 (0.78) | 0,73 (1.25) | 0.76 |
| Habitual PA practice, score | 2.19 (0.64) | 1.94 (0.51) | 0.14 |
|  |  |  |  |
| Sports practice, n (%) |  |  |  |
| Yes | 51 (17.7) | 3 (23.1) | 0.62 |
| No | 237 (82.3) | 10 (76.9) |  |
| Mother's educational level, n (%) |  |  |  |
| Secondary education incomplete | 13 (13.7) | 0 (---) | 0.89 |
| Secondary education complete | 3 (10.5) | 0 (---) |  |
| High school | 59 (47.6) | 2 (100) |  |
| Graduated | 24 (19.4) | 0 (---) |  |
| Not informated | 11 (8.9) | 0 (---) |  |
| Father's educational level, n (%) |  |  |  |
| Secondary education incomplete | 24 (21) | 0 (----) | 0.40 |
| Secondary education complete | 18 (15.8) | 2 (50) |  |
| High school | 40 (35.1) | 2 (50.) |  |
| Graduated | 13 (11.4) | 0 (----) |  |
| Not Informated | 18 (16.7) | 0 (----) |  |

SD, standard deviation; n, sample;**BMI:** Body mass index; WC: waist circumference; **PHV:** Peak height velocity, **PA:** Physical activity. * P<.05 significant difference between groups.
