# Supplementary figures and images for: Impact of the ActTeens Program on physical activity and fitness in adolescents: a cluster randomized controlled trial
Source: BMC Pediatr. 2024 Jul 11;24:447. doi: 10.1186/s12887-024-04922-9 (PMC11238359; doi:10.1186/s12887-024-04922-9)

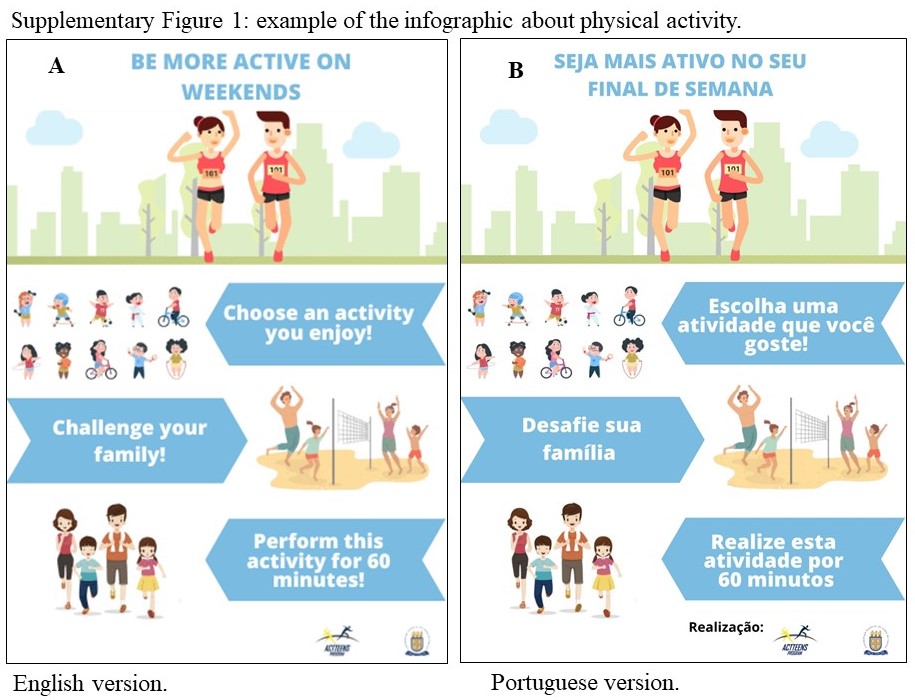


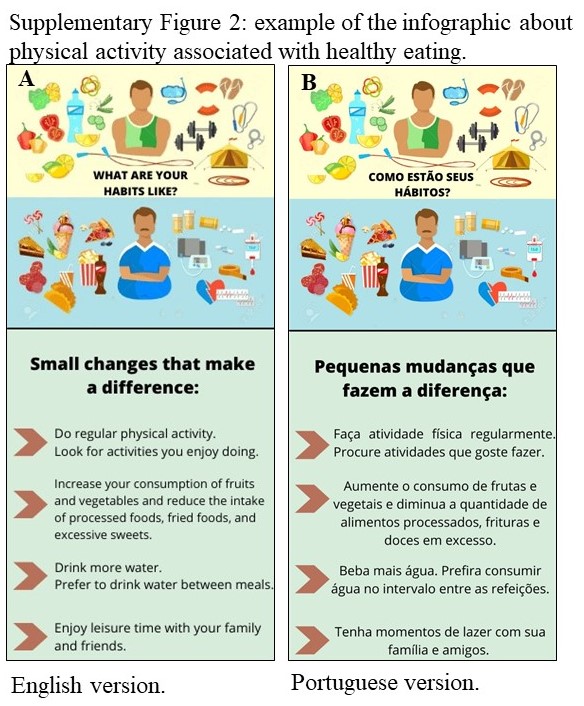


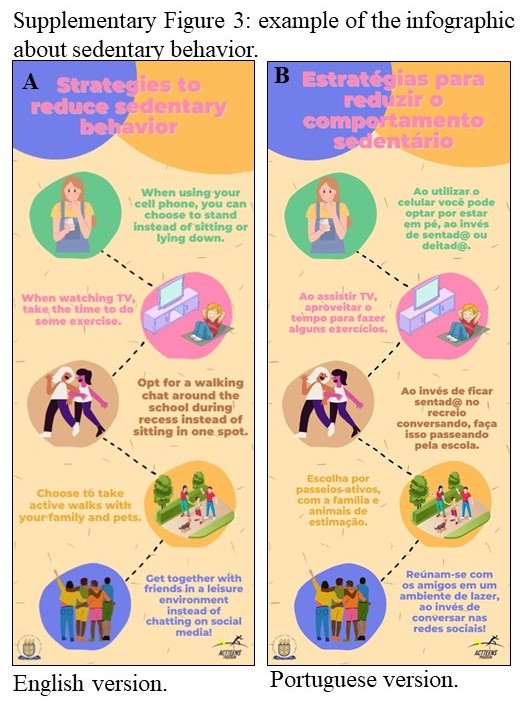

Supplement: Supplementary file 4 — Supplementary Material 4 [file 12887_2024_4922_MOESM4_ESM.docx]
